# Physiotherapy students’ perceptions of the dual role of the clinical educator as mentor and assessor: Influence on the teaching–learning relationship

**DOI:** 10.4102/sajp.v73i1.349

**Published:** 2017-07-27

**Authors:** Ilse S. Meyer, Alwyn Louw, Dawn Ernstzen

**Affiliations:** 1 Centre for Health Professions Education, Division of Physiotherapy, Faculty of Medicine and Health Sciences, Stellenbosch University, South Africa; 2 Centre for Health Professions Education, Faculty of Medicine and Health Sciences, Stellenbosch University, South Africa

## Abstract

**Background:**

Clinical education is widely considered to be the cornerstone of health care professionals’ education. Clinical educators (CEs) fulfil many roles and act as both mentors and assessors in the learning process of students’ undergraduate health care professions education. However, changing from being a mentor to being an assessor may present particular challenges for both the CE and the students.

**Objective:**

To explore students’ perceptions of how the dual role of a CE as mentor and assessor influenced the teaching–learning (T-L) relationship.

**Method:**

A qualitative descriptive study, involving seven individual semi-structured interviews and two focus group discussions, was conducted with students in the Division of Physiotherapy, Stellenbosch University. A contextualised interpretive content analysis was used to analyse the data. By following an iterative process, themes were identified and categories were reviewed and refined.

**Results:**

Challenges were experienced when CEs had to act and change as both mentors and assessors to the needs of the students. This influenced the T-L relationship and consequently impacted the learning of students. The expectations of students and CEs were often not fulfilled. Contradictions were disclosed regarding the dual role of CEs.

**Conclusion:**

The findings of the study, grounded in the perceptions and experiences of students on the dual role of the CE, are highlighted. It is important to consider the challenges that the students face in order to minimise any negative effects these challenges could have on students’ learning processes.

## Introduction

Clinical education is widely considered to be the cornerstone of health care professionals’ education and is described as the key component that prepares health professionals for practical experiences (Kilminster et al. 2007; Laitinen-Väänänen 2008). In the clinical environment, clinical education remains a powerful teaching context as it provides an authentic experience during which students are actively engaged in the learning process (Griffiths & Ursick 2004; Ker, Cantellon & Ambrose 2008; Webb 2004). Although the recent approach to clinical education has shifted from a more traditional ‘teacher-centred’ to a ‘student-centred’ approach and has improved the overall effectiveness of this type of training, problem areas remain. In this study, the teaching–learning (T-L) relationships between clinical educators (CEs) and students from the Division of Physiotherapy, Stellenbosch University (SU), were evaluated. Problems arising from the dual role of the CE as both mentor and assessor to students and the influence that the students’ perceptions of these roles have had on the T-L relationship were identified.

Harden and Crosby (2000) and Ernstzen, Bitzer and Grimmer-Somers (2009) acknowledged that CEs fulfil many roles, such as curriculum planners, information providers, role models and resource developers. CEs at SU are also responsible for fulfilling some of these roles. Furthermore, CEs at SU act as both mentors and assessors in the learning process of students’ undergraduate health care professional education.

During their clinical education, physiotherapy students at SU acquire clinical skills, values and attitudes through individual or group supervision sessions while interacting weekly with their CEs at a variety of clinical placements through which they rotate. The focus of the approach to learning is on student-centredness where students identify their own needs for learning and are, therefore, actively engaged in their own learning process. This is done via a strengths, weaknesses, opportunities and threats (SWOT) form where they can construct, discover and transform knowledge during clinical education (SU 2012). Affective, psychomotor and cognitive skills are combined in this socio-emotional environment (Boud & Falchikov 2007). A cycle of action and reflection (Kolb 1984; Schön 1995) centred in communities of practice (Wenger 1999) is followed at SU during discussion sessions between students and CEs. These discussion sessions assist to facilitate learning through reflection during their clinical education, where the students feel they are equal partners. These sessions facilitate socialisation and ensure that learning occurs, personal identities are developed and personal and professional growth take place (Laitinen-Väänänen 2008).

According to Entwistle and Peterson (2004), there are three different approaches to students’ learning, namely a surface, a strategic and a deep approach to learning. The challenge for CEs is to facilitate students to engage in a deep approach towards learning by encouraging and motivating them. The deep approach towards learning fosters an active participant in the learning process where knowledge is created to give personal meaning to the student. This can be true of a strategic approach towards learning, but the difference lies in the motivation to learn. To be personally engaged in the learning process is to become intrinsically motivated. Rose and Best (2005) highlighted that motivation is essential for students’ learning. Students who use a surface or strategic approach towards learning have different intentions. With a surface approach, students are extrinsically motivated, while with a strategic approach, their intentions are to achieve the highest possible grades. The experience in the clinical environment may facilitate a change in the learning approach of students (Best, Rose & Edwards 2005). According to Vygotsky’s principles (Vygotsky & Cole 1978), the learning zone defines the zone of actual development in which students function on a level without assistance where they are active and responsible. Working within this zone, the students have already mastered the skills expected of them during activities in clinical education. The CE can help students to gain new knowledge, from a prior knowledge base, which is appropriate for their level of comprehension. For learning to be effective, the zone of absolute development of the student is established as students need to disclose their limitations in order for the CE to facilitate their learning towards the zone of proximal development. This zone is the area where learning actually takes place (Vygotsky 1962).

As mentors, CEs should facilitate students’ learning by investing time and energy, offering support to students and taking an interest in them. CEs should guide the students, share their own experiences with them, give honest feedback and encourage students’ self-development. They should provide appropriate knowledge-based information to encourage students to build their confidence and to link theory to practice (Morton-Cooper & Palmer 2000). The mentoring relationship that is established between a student and CE is seen, therefore, as a powerful tool for advancing clinical skills (Ezzat & Maly 2012).

In the final week of a clinical rotation, the CE takes on the role of an assessor of clinical competence. The role changes to that of a judge and involves assessing students’ progress and their performance, which includes skills, attitudes and behaviours, and reporting on them (Price 2004). The CE becomes professionally accountable to society, professional bodies and to patients for their judgement when assessing if students are ‘fit for purpose’ (Wass et al. 2001). According to Kilminster et al. (2007), assessment practice should protect the public as well as foster habits of learning and self-reflection, and should drive institutional change. The success of students’ learning depends, however, on the manner in which the teaching, learning and assessment activities are conducted as well as the T-L relationships (Vermunt 2005). Students’ learning occurs not only from what is taught but also substantially from the mentor–student relationship. Students identify relationships with their CEs as critical to the success of their learning experience (Miller 2012). Several researchers agree that the T-L relationship between a CE and a student is probably the most important aspect of clinical education as the CE provides support and a sense of belonging (Gallagher et al. 2012; Kilminster et al. 2007). The quality of the T-L relationship, however, will affect students’ confidence and willingness to engage actively in the learning process (Delany & Bragge 2009).

Having to change from being a mentor to being an assessor, however, may present particular challenges for both the CE and the students. These challenges may affect the quality of the relationship between a CE and a student and may seriously affect learning, particularly if there is any disparity in either party’s expectations (Hodges 2009). It was necessary, therefore, to determine how these challenges might affect the T-L relationship between the CE and students.

The study aimed to:

- explore the students’ perceptions of how the dual role of CEs as mentors and assessors of students influences the T-L relationships
- evaluate these perceptions
- find possible answers to problem areas in order to improve the quality of students’ learning.

The objectives of this study were to:

- determine how the students constructed meaning from their clinical education
- explain their experiences of clinical education with a view to improve the quality of their learning.

## Study design

### Study approach and method

This study followed a qualitative research approach, with an interpretivist paradigm, which was conducted by means of a phenomenological inquiry (Maree 2007). This approach is underpinned by collecting information through observation and interpretation from the participants in the study. Observations and interviews are the key data collection methods within phenomenological studies. In this way, meaning could be constructed by judging the information and analysing it in context. The investigation was based on third- and fourth-year students’ experiences at the Division of Physiotherapy, SU.

### Participants and recruitment procedures

In-depth, semi-structured, individual interviews were arranged with four fourth-year and three third-year undergraduate physiotherapy students. In addition to these, two students focus groups were conducted, consisting of eight third-year and eight fourth-year students. Focus group discussions were included to allow the students to share their experiences and to trigger concepts mentioned by fellow students that might otherwise not have emerged. A focus group provides the opportunity for participants to build upon each other’s contributions and for new understanding to further develop (Wibeck 2007). Triangulation is a method used by qualitative researchers to check and establish validity, by analysing a research question from multiple perspectives (Terre Blanche et al. 2006). Purposive sampling was conducted using a sampling framework. In order to ensure sample diversity, the key sampling criterion was participants’ clinical assessment results at the end of their first rotation. An equal number of students from third and fourth year who scored low, average and high percentages in their first clinical competency assessment were eligible to participate. The choice of participants was made arbitrarily by an administration officer and covered the noted dimensions of diversity regarding their results within the two groups. This ensured a wide perspective of the students’ experiences of the T-L relationship. Emails were sent to the selected students inviting them to participate. Interviews and discussions were scheduled over a period of two months accordingly. The students’ ages ranged between 21 and 25 years. They were all female. The individual interviews were conducted in Afrikaans and English, according to the language preferences of the participants. All interviews took place at the Division of Physiotherapy at the Faculty of Medicine and Health Sciences (FMHS), SU. Participation was voluntary, and written informed consent was obtained from all participants. The participants were assured of the confidential handling of the data.

An interview discussion schedule was developed by the first author, who provided guidelines for defining the line of enquiry. This discussion schedule was used for both individual and focus group interviews. The discussion schedule assisted the interviewer in maintaining focus and ensuring coverage of all important issues. The questions were open-ended questions based on the aim of this study as well as on aspects from the literature that appeared to have an impact on the T-L relationship. The first author and interviewer discussed the interview questionnaire to ensure that it could be used effectively.

### Data management and analysis

The interviews were recorded on a digital voice recorder, downloaded onto a computer and protected by password. A unique serial number was allocated to each recording and copied onto a computer flash disk. An independent assistant transcribed the recorded interviews and saved them on a secured computer for the research period. The transcribed interviews were made available for member checking.

A contextualised interpretive content analysis, as defined by Miles and Huberman (1994), was used to analyse the data. The verbatim transcripts were studied in order to become familiar with the contents. Microsoft Office OneNote 2007 was used to code transcripts, notes and written analytic memoranda. The data were coded by identifying patterns present. The patterns that emerged from the data were arranged into themes and entered into a codebook. The themes and the developing categories were reviewed and refined. An iterative process between the literature and the research question was followed during the course of the study (Kelly 2009). To add to the trustworthiness of the findings, the supervisors of this study checked the themes and categories against the transcriptions of the interviews (Lincoln & Guba 1985). Rather than ascribing individual comments to a particular participant, an inclusive data set was used in the results below.

### Ethical consideration

Ethical approval for the study was obtained from the Human Research and Ethical Committee (HREC), Faculty of Medicine and Health Sciences (FMHS), SU (Protocol number: S12/11/289), and from the Institutional Research and Planning Division (IRP) at SU. Permission to conduct the study was obtained from the chairperson of the Division of Physiotherapy’s undergraduate programme committee and the Head of the Division of Physiotherapy at SU. The first author was employed as a CE and clinical coordinator at the Division of Physiotherapy, SU, during the study and consequently fulfilled the dual role of both mentor and assessor for fourth-year undergraduate physiotherapy students. The first author was also the CE for a third-year clinical rotation in 2012, which meant that the 2013 cohort of fourth-year students had prior clinical education experience with her. Therefore, as a CE, the first author was involved with the participants, their experiences, their biases and interests. The process of gathering the data was outsourced to an independent and experienced interviewer from the Centre for Health Professional Education at SU in order to enhance the credibility of the study. This was to ensure that the first author as a CE had no influence on the gathering of the data.

## Results

Three main thematic categories were identified within the data:

- the challenges of the dual role within the T-L relationship
- expectations of the T-L relationship
- students’ preferences regarding the dual role of the CE.

From these identified categories (see Table 1), three main themes emerged from the data.

**TABLE 1:** Themes and categories that emerged from the data.

Themes	Categories
Challenges	Inconsistencies Subjectivity Conflict Failing students Intimidation Feedback Shortcomings
Expectations	Participants and their relationship
Preferences	Roles Relationship

*Source*: Authors’ own work

### Theme 1: Challenges

The dual role was perceived by students as challenging within the T-L relationship. These dual role challenges impacted negatively on the learning experiences of students.

*Inconsistencies*: Participants in this study experienced inconsistencies in the behaviour and attitudes of a CE when having to change from the role of mentor to assessor. CEs would, for example, prompt a student during a formative assessment while not doing it during a summative assessment. Another example is where CEs have different expectations from students during a summative assessment for the same clinical case. This caused students to perform strategically according to the preference of the CE during assessments. The perceived inconsistencies caused disharmony within the T-L relationships as well as in the assessment process. Participants found it difficult to adjust to the change in the role of the CE from an advisor to an evaluator or judge. This confused the students and deflected their focus away from the assessment event towards trying to satisfy the CE whom they had come to know as their advisor, as is evident in the quotation below.

‘Some clinical educators are too friendly and too generous when they mentor and then they truly swap when it comes to assessing. They’re strict and then you get two different views of the person.’ (Participant 10, female, physiotherapy student)

*Subjectivity*: CEs were prescriptive when they assessed the students’ performances. Limited space for students’ initiatives was conceded during assessments.

‘When they’re marking, the clinical educators are very subjective instead of being objective. They [*CEs*] are marking you during an assessment, so now you would rather be on the lookout for what she would expect you to do. So you adapt towards their way of thinking, as they will be assessing you. If the clinical educator asked me to focus more on specific techniques, I would make sure I also do it during the assessment as I realised that she would also, during the assessment, want me to focus on these.’ (Participant 9, female, physiotherapy student)

*Conflict*: Furthermore, conflict between the students and the CEs arose from different situations. Conflict in this context is seen as a situation in which there is disagreement between participants and CEs in the T-L relationship. Potential conflict situations developed when CEs failed students during clinical performance assessments. The students found it traumatic and said that it could affect the T-L relationship by inhibiting open communication and trust as they lost their confidence to participate.

‘I felt really, really, terrible after failing my clinical block assessment. I nearly had a breakdown. I also had personal problems. … My self-confidence was so low.’ (Participant 6, female, physiotherapy student)

*Intimidation*: Participants sometimes perceived CEs to be intimidating. They felt uneasy about asking questions or revealing their weaknesses to CEs. Some CEs dominated the learning sessions without considering students’ feelings.

‘Sometimes clinical educators can be quite short or brief or get annoyed with you asking questions or don’t like explaining things twice. That makes us nervous to ask questions or feel more stupid for not understanding when they’ve explained the first time while you just understand a different way of explaining. You’re going to set yourself up for failure, disappointment from the educator. Often we are reluctant to ask questions as we feel intimidated and you … hmmm don’t want to sound stupid in front of them, because at the end of the day, they’re the people that will be assessing you. You are scared that they will remember, while assessing you, that you gave a silly answer, so you don’t want to be honest in saying what you think. So I don’t feel like telling them what I don’t know.’ (Participant 10, female, physiotherapy student)

*Feedback*: Participants valued appropriate and adequate feedback during clinical rotation. Some participants reported a lack of adequate feedback. This could cause confusion and uncertainties about the quality of their clinical practice. Participants indicated that CEs’ attitudes and responsibilities played an important role in developing a favourable learning environment.

‘Yes, feedback, it was something I really wanted on my clinical block. Sufficient feedback, it is something I value a lot, but I never received that feedback.’ (Participant 7, female, physiotherapy student)

*Shortcomings*: The shortcomings in CEs’ attitudes and responsibilities could affect the T-L relationships negatively. Participants found some CEs impatient, unhelpful, lacking passion and at times unapproachable.

‘Sometimes it happened that a clinical educator is somewhat impatient if you don’t know the answers. It’s so demoralising when you see a clinical educator who looks like they don’t want to be there. A CE should be passionate and determined. I think my CE didn’t play the role that she needed to play. If I had that type of guidance, I would have passed. I told my CE, but I never got the help I needed. I really think we needed that guidance, especially for our first block. There wasn’t a time where we could say, “Can I please have you to come with me to one of my patients and help me?” They’re [*CEs*] not very approachable. That’s the challenge. You can’t confide in them like you want to.’ (Participant 4, female, physiotherapy student from Focus Group 2)

### Theme 2: Expectations

Participants pointed to the need for effective relationships where good communication, mutual respect and trust were present. The importance of mutual goals, namely to reach the learning outcomes of the clinical rotations, was emphasised. Participants expressed their views on what they expected from the T-L relationship as evident in the following quotation.

‘I think communication is a big … a big factor in a relationship. Planning and structure from both sides are important. Plan the goals and reciprocal respect to reach the goals. Very relaxed and a simple relationship, to feel comfortable to ask questions. You need to feel comfortable with the person. There should be trust and a good understanding between you, then we know what to expect from each other.’ (Participant 12, female, physiotherapy student)

A comfortable learning atmosphere was indicated as an important prerequisite for the development of a positive T-L relationship. This was important to ensure open conversations, safe environments and comfortable relationships where students could feel free to discuss their weaknesses and strengths and to ask questions. The participants highlighted the need for a well-structured, comfortable and relaxed learning atmosphere, which allows enough space for both parties to build a T-L relationship that could withstand the challenges inherent in the dual role of the CE as mentor and assessor.

### Theme 3: Preferences

Despite the immense challenges that are part of the dual role of the CE as mentor and assessor, some participants indicated a preference for the dual role. The dual role enables both parties to get to know one another, allowing time to develop a T-L relationship and to clarify expectations. Knowing who the assessors were going to be, the participants found that this could be beneficial to them as they could adapt to their assessors’ preferences and expectations.

‘I think it’s quite important that they’re the same person, because when they mentor you, you kind of work at what they want and how they want it done. They have seen the progress that you’ve made and now they can put it all together into your final assessment. You adapt towards their way of thinking, as they are assessing you at the end of the block. You observe to see what they prefer, how they expect you to do things and I think it helps during the assessment session. If you know this person prefers a certain way of doing things, then you rather do it accordingly, because she will assess you.’ (Participant 7, female, physiotherapy student from Focus Group 1)

Some participants were adamant that external assessors would be stressful for them as they would not know what the assessors’ expectations and preferences were and they would have no relationship with them.

‘If an external examiner arrives, you experience anxiety, as this person doesn’t know you. No one can treat a patient effectively when they feel anxious.’ (Participant 7, female, physiotherapy student from Focus Group 2)

In contrast, a subgroup of participants preferred a different assessor. The benefit of an external person was based on the perceived objectivity that this person could add to the assessment process.

‘I think the dual role of a clinical educator is a bit of a bad idea. An external examiner, you know, comes with a different view. She doesn’t know you at all, so you can expect that there’s nothing about the block that she keeps in mind. You have to know your story, but for me it felt like more … hmmm … she’s more objective. I feel the system, as it is, seems a bit subjective.’ (Participant 8, female, physiotherapy student)

Some participants perceived the dual role of a CE as both a positive and a negative experience.

‘I think it’s positive and negative. Positive, because you’re getting an objective person, who has not been with you each day, so, she’s new. Negative, because you’re not building up a relationship with your clinical educator.’ (Participant 7, female, physiotherapy student)

Some participants preferred a relationship where they were seen as equal partners.

‘So for me, they’re seeing me as a colleague, to challenge me to develop. It’s like saying, I stand by you.’ (Participant 7, female, physiotherapy student from Focus Group 2)

The participants’ preferences indicated that they were aware of the advantages as well as the disadvantages of the dual role of the CE as mentor and assessor.

## Discussion

In this study, the T-L relationships between CEs and students were evaluated against the students’ perceptions of this role and how these perceptions have impacted on the T-L relationship. Good communication skills were necessary where parties shared power in communities of practice, and showed mutual respect and trust in the T-L relationship. Students expected the relationship to be reciprocal, relaxed and open, so that they would feel comfortable asking questions. These findings resonate with findings in previous studies (Buccieri, Pivko & Olzenak 2013; Oyeyemi et al. 2012). If these expectations were not addressed, the T-L relationship was affected negatively.

The findings indicated that the dual role of the CE had a strong influence on the T-L relationship and that this relationship was affected by a range of challenges, expectations and preferences. The participants identified factors that challenged the T-L relationship. These included inconsistencies in attitude and behaviour, a lack of constructive feedback, subjectivity of CEs, anxiety and confusion when students failed, intimidation of students and expectations that were not met. These factors affected the T-L relationship and were detrimental to students’ learning.

The perceived inconsistencies of the CEs were considered to be a challenge that negatively influenced the T-L relationship. These inconsistencies mainly related to the change from mentor to assessor where students perceived a change in the behaviour and attitude of some CEs. Describing the assessment process, students acknowledged that they were sensitive to the fact that their mentors were now acting as their assessors as the personal dynamics between the two role-players changed. However, students had difficulty in adjusting to this shift in role and felt that this had confused them and it disturbed the harmony in the T-L relationship. The participants found that as mentors, the CEs were usually friendly and helpful. When the CEs changed to the role of assessor, however, the behaviour and attitude of some of the CEs changed. Some were also inconsistent in the ways they wanted students to perform during clinical assessments. These inconsistencies have previously been found to influence the reliability and validity of the assessment procedures, as confirmed by Gravett and Geyser (2004). Students then acted as strategic learners, aiming to please the CE during the assessment process. Students performed as they thought the CE as assessor would like them to perform. Inconsistencies arose between what the student perceived that the assessor would ‘want’ and which interventions, during the assessment, could be applied.

Furthermore, during this study, participants reported that the ability of some CEs to remain objective during the assessment process was perceived to be hampered by the fact that the CE had to function both as mentor and assessor. An awareness of subjectivity in CEs was a major challenge during the process of assessment. Evidence in a study done by Alexander (1996) suggested that assessors make subjective judgements about students and that these judgements influence assessment grades.

The perceived bias of some assessors caused problems where assessment using observation occurred. The findings indicated that the participants’ perceptions of the subjectivity of some CEs contributed to students’ unwillingness to reveal their own limitations and weaknesses. This aspect was, therefore, counterproductive to achieving the learning outcomes – but more importantly, counterproductive to learning. Gilbert and Malone (1995) as well as Borrell-Carrió and Epstein (2004) state that bias can lead to inaccurate assumptions being made. In summative assessments, high reliability is necessary to judge students as the results are used for selection and criteria processes (Baartman et al. 2007; Roberts et al. 2006). The lack of efficient objectivity by CEs in the performance assessments of students could lead to unreliable and invalid assessment procedures. In contrast, however, Van der Vleuten and Schuwirth (2005) state that reliability is not subjected to objectivity, but rather to the amount of sampling across different contexts and assessors.

Students felt dissatisfied when feedback was inadequate. The findings of the study confirmed that participants found the overpowering attitudes of CEs intimidating. The participants experienced conflict when CEs suppressed them. They tried to maintain the social harmony within the T-L relationship by not causing conflict and this prevented them from expressing their opinions, or asking questions, which led to a lack of trust and transparency. Lee, Cholowski and Williams (2002) mentioned that CEs sometimes claim that they put the students at the centre of learning in practice, but in reality, they do not. Intimidation of students contributed to a feeling of alienation, which impeded communication in the T-L relationship. The participants commented that some CEs did not give adequate, appropriate, constructive and timely feedback. This confirmed the findings of a study conducted by Molloy (2004) regarding students’ experiences of feedback sessions. In this study, participants experienced feedback sessions as an asymmetrical process. The feedback sessions illustrated the power imbalances that existed between some students and CEs (Molloy 2004). According to Ratner (2000), students can see their CE as an authority figure displaying considerable power in this relationship. Bloxham and Boyd (2007) mentioned that students need feedback about their performances in order to improve their future learning. CEs should, therefore, be aware of the value of constructive feedback in the students’ learning processes.

Some participants reported that the expectations they had of CEs were sometimes not fulfilled. Some CEs were not readily available and approachable to discuss their problems and often they did not feel comfortable enough to ask questions. Buchel and Edwards (2005) stated that CEs should be readily available when help is required and that they should ensure that a safe, non-judgemental and non-threatening learning environment is established.

A number of participants preferred that the CE acted both as mentor and assessor in the T-L relationship. The CE and the students got to know one another well, even on a personal level. Researchers have consistently found personalisation from students’ perspectives as an important component, indicating the concern students have regarding their own welfare (Brown et al. 2011; Smedley & Morey 2009). The participants indicated that knowing who the assessors were going to be was beneficial to them as they could adapt to their assessors’ preferences and expectations. This again encouraged students to adopt a strategic approach to learning, and students preferred the dual role in order to gain adequate pass rates. Such an approach was counterproductive as the students used extrinsic motivation to attain pass marks for the assessment, while intrinsic motivation ideally leads to a deeper learning approach. Intrinsic motivation will most probably lead to a deep approach towards learning (Rose & Best 2005). The challenge is therefore to shift the focus towards a learning-centred approach where the focus is to learn from the experience. According to some of the participants, the presence of an external assessor caused stress and made them anxious. Anxiety experienced by students can push students towards a surface approach to learning (Mayya, Krishna Rao & Ramnarayan 2004). In contrast, however, some students preferred a different assessor who would demonstrate a more objective view during clinical assessments. Participants were aware of both the advantages and disadvantages of the dual role of the CE as mentor and assessor.

A limitation of this study, as with any qualitative study, is the fact that the findings can only be generalised in similar contexts rather than encompassing the broader, structured context of the clinical environment. A further limitation involved the use of interviews as the only source of data collection and analysis. This study focused only on the students’ perceptions. The CEs’ perceptions, which were also identified during this comprehensive study, will be presented in a follow-up article to allow for a more holistic view on this topic.

## Conclusion

It became apparent that the dual role of the CE influenced the T-L relationship. The findings addressed the views of the students on the dual role of the CE as mentor and assessor and the performance standards that were expected from both roles. The social forces that existed in the T-L relationship had a significant impact on students’ learning. Disparities arose when CEs acted as both mentors and assessors, which caused disharmony in the T-L relationship and thereby affected students’ learning. These disparities were identified as challenges, expectations and preferences. It was important to consider the challenges that the students faced in order to minimise any negative effects these challenges could have had on the students’ learning processes. Students had mixed feelings about the dual role of the CE. If the expectations of both parties were met, it could lead to the transformation of the T-L relationship. A learning-centred paradigm could be established, driven by open communication, as students and CEs then can collaborate as equal partners in communities of practice.
